# Motivational neurobehavioral abnormalities under a naturalistic goal-conflict task in patients with premenstrual dysphoric disorder

**DOI:** 10.3389/fpsyt.2026.1776826

**Published:** 2026-06-08

**Authors:** Yulia Lerner, Michael Krasnoshtein, Miki Bloch, Michal Tevet, Maayan Doron, Tal Gonen, Talma Hendler, Oren Tene

**Affiliations:** 1 Sagol Brain Institute, Tel Aviv Sourasky University Medical Center, Tel Aviv, Israel; 2 Gray Faculty of Medical & Health Sciences, Tel Aviv University, Tel Aviv, Israel; 3 Sagol School of Neuroscience, Tel Aviv University, Tel Aviv, Israel; 4 Psychiatry Division, Tel Aviv Sourasky University Medical Center, Tel Aviv, Israel; 5 School of Psychological Sciences, Tel Aviv University, Tel Aviv, Israel

**Keywords:** affective disorders, approach-avoidance behavior, fMRI, mesolimbic pathway, motivation, reward circuitry

## Abstract

**Introduction:**

Motivation drives goal-directed behavior, often requiring individuals to approach rewards while facing potential punishment (i.e., goal-conflict). Premenstrual dysphoric disorder (PMDD) is marked by emotional and cognitive impairments that may affect motivational functioning. While emotional deficits in PMDD are well-known, their impact on motivation under goal-conflict remains unclear. This study examined neurobehavioral indications for motivational deficits in PMDD during naturalistic goal-conflict and their relation to symptom severity.

**Methods:**

Fifty-one women with regular menstrual cycles (28 with PMDD, 23 healthy controls) completed a computer-based approach-avoidance task during fMRI scanning in the luteal phase. The task included high and low goal-conflict (HiGC or LoGC) conditions, where rewards were threatened by punishment. Neural activity in mesostriatal reward-related regions – the ventral striatum (VS) and ventral tegmental area (VTA) – was analyzed using general linear models. Symptom severity was assessed using the Premenstrual Tension Syndrome Observer Rating Scale (PMTS-OR).

**Results:**

Compared with healthy controls, participants with PMDD were less able to increase approach behavior as conflict decreased, resulting in fewer approach events under LoGC condition, while no group differences were observed under HiGC. Additionally, within the PMDD group, greater symptom severity (depression and anxiety) was associated with lower approach behavior under HiGC conditions. fMRI analyses revealed diminished activation in the VS and VTA under HiGC in the PMDD group, suggesting reduced conflict-related engagement of reward-related circuitry.

**Conclusion:**

PMDD is associated with impaired motivational adaptation across conflict levels, reflected in a blunted increase in approach behavior under conditions of lower risk. Diminished conflict-related activity in reward-related mesostriatal regions (VS, VTA) may underlie this deficit. These findings highlight the potential role of mesolimbic circuitry dysfunction in PMDD and point toward reward processing pathways as candidate therapeutic targets.

**Trial registration number:**

NCT02448836, ClinicalTrials.gov.

## Introduction

Motivation is a fundamental driver of human behavior, essential for productivity and achievement. Motivated behavior is expressed as the approach toward or avoidance of goals, driven by the desire for rewards or the threat of punishment (1). The brain’s reward system guides behavior toward stimuli of positive value and away from negative ones. A key challenge in real-life situations is the approach-avoidance conflict, where rewards and punishments coexist, often stemming from stimuli with mixed or ambiguous value (2). Successfully navigating these conflicts requires adaptive motivational behavior, which plays a crucial role in balancing the desire toward approaching a reward and avoiding punishment in a constantly shifting environment (3).

Motivational behavior is shaped by both physiological and psychological states, with dopamine playing a central role. Over the past two decades, neuroimaging studies have consistently demonstrated that the dopaminergic mesolimbic pathway regulates reward-driven behavior in humans (4). Additionally, a study conducted in our lab identified neural patterns underlying motivational behavior, highlighting the interaction between approach behavior and individual goal-conflict tendencies (5). Specifically, increased neural activity has been observed in dopamine-rich regions such as the substantia nigra (SN) and ventral tegmental area (VTA), along with dopamine release in the ventral striatum (VS) and nucleus accumbens (NA) (6–8). These regions are key to reward processing and motivational behavior. The orbitofrontal cortex (OFC) and ventral medial prefrontal cortex (vmPFC) further enhance motivation by directing attention toward pleasurable rewards and regulating decision making (9, 10).

Despite substantial research on the nature of motivation and the neural mechanisms underlying incentive motivation, relatively few studies have explored how these processes are altered in the context of psychiatric disorders. Motivational deficits are common across various psychopathologies, including depression and substance use disorders (11). Given the central role of the dopaminergic system in mood regulation (12), disruptions in this system may significantly contribute to functional impairments across different psychiatric conditions.

One such condition is Premenstrual Dysphoric Disorder (PMDD), which affects up to 8% of women of reproductive age (13). Hormonal fluctuations, particularly elevated estrogen and progesterone during the luteal phase (the second half of the menstrual cycle, occurring after ovulation and before the start of menstruation), influence both emotional and motivational processes in PMDD (14). While emotional impairments during the luteal phase are well-known (15), findings on goal-directed motivation remain mixed (16). Women with PMDD often report reduced work productivity during this phase (17), which may be linked to disruptions in motivational processes. Indeed, impaired goal-directed behavior and reduced reward sensitivity are known to negatively impact daily functioning and overall quality of life (18).

The link between dopamine levels and mood changes suggests dopamine’s potential role in the development of PMDD (19). While the exact role of dopamine in PMDD remains unclear, hormonal fluctuations may alter the brain’s reward system, leading to motivation deficits and cognitive impairments during the luteal phase. Motivational deficits in conditions such as depression and schizophrenia have been associated with dopamine dysregulation (20, 21). Disrupted dopamine signaling reduces the motivation to seek rewards (22) and is also linked to stress-induced depression (23). Similarly, altered dopaminergic activity during the luteal phase may underlie motivational deficits in PMDD, including reduced goal-directed behavior and reward sensitivity.

Animal and human studies have consistently highlighted the key role of the VS (24–26) and VTA (27, 28) in reward processing and motivation, mediated by the mesostriatal dopaminergic pathway. Gonen et al. (29) demonstrated that approach behavior under goal conflict – approaching a reward while facing the risk of punishment – requires heightened incentive motivation and is associated with increased activity in both the VTA and VS. Building on these findings, the current study used the same computer-based game to assess motivational behavior in patients with PMDD (during the luteal phase) compared to healthy controls. During an fMRI scan, participants played a game in which monetary rewards were threatened by punishments, creating conditions of high and low goal conflict (HiGC and LoGC, respectively), corresponding to more or less risk of punishment while approaching a reward. Unlike previous PMDD studies that primarily focused on negative emotional responses (30), this study specifically examined deficits in reward processing.

We hypothesized that greater affective symptom severity (e.g., depression, anxiety, anger, emotional lability) would be correlated with reduced motivation to approach rewards under risk, as reflected by fewer approach behaviors in HiGC situations. At the neural level, we expected to observe lower activity in reward-related regions during these periods, indicating diminished incentive motivational processing in patients with PMDD.

## Methods

### Participants

A total of 51 women with regular menstrual cycles were recruited and screened by a senior psychiatrist at the Psychiatry Division at Tel Aviv Sourasky University Medical Center (TASUMC). The study included 28 women diagnosed with PMDD (ages 21–46; mean age = 33.4 ± 5.9) and 23 healthy controls (ages 26–47; mean age = 32.1 ± 7.9). Three participants with PMDD and three healthy controls were excluded from the analyses due to excessive head motion (>3 mm) during the fMRI scans. All participants had normal or corrected-to-normal vision, were right-handed, and reported no history of head trauma or neurological disorders.

#### Screening and diagnostic confirmation

Initial screening was conducted using the Premenstrual Symptoms Screening Tool (PSST; 31) to identify suspected PMDD and ensure that healthy controls did not meet PMDD criteria. PMDD diagnoses were subsequently confirmed based on DSM-5 criteria (American Psychiatric Association, 2013) and verified through monitoring over at least two menstrual cycles using the Daily Record of Severity of Problems (DRSP; 32). Ovulation and luteal phase onset were confirmed through a standard commercial urine test (*Meditest*, Nantong Egens Biotechnology Co., Ltd.).

Exclusion criteria included moderate to severe polycystic ovary syndrome, use of a hormonal intrauterine device, initiation of hormonal or antidepressant treatment within the past 3 months, or drug abuse (excluding nicotine) in the past 3 months. Participants were also free of major depressive episodes or active anxiety disorders at the time of study entry. The Premenstrual Tension Syndrome Observer Rating Scale (PMTS-OR; 33) was used to assess ongoing PMDD symptoms. In addition, the Clinical Global Impressions - Severity of illness (CGI-S; 34) and Big Five Inventory assessing personality variables (BFI; 35) were administered.

Detailed socio-demographic information, clinical characteristics of the participants, and psychiatric evaluation scores are provided in **Table 1**. Group differences in Age and Education were assessed using the Mann-Whitney U test and the Kolmogorov-Smirnov test. The Mann-Whitney U test indicated a significant difference in central tendency for Education between the groups (U = 341.5, p = 0.016), which was insignificant for Age (U = 202.5, p = 0.38). The Kolmogorov-Smirnov test was not significant, suggesting no strong global distributional differences. Group differences in other variables were examined using Fisher’s exact test, which revealed no significant differences between groups. Notably, smoking status and BMI were balanced between groups, which is important given their potential influence on VTA activity.

**Table 1A:** Socio-demographic characteristics of all participants in the study.

Metric	Patients with PMDD (N = 25)	Healthy Controls (N = 20)	Between-Group Comparison *p*-value
Age (years)	33.4 ± 5.9	32.1 ± 7.9	*p = 0.5* [KST] *p = 0.38* [MWU]
Education (years)	15.3 ± 2.9	17.5 ± 2.8	*p = 0.4* [KST] *p = 0.016* [MWU]
Marital Status (in relation/not)	14/11	13/7	*p = 0.5* [FT]
Children (y/n)	10/15	6/14	*p = 1.0* [FT]
Employment (y/n)	20/5	20/0	*p = 0.1* [FT]

KST, Kolmogorov-Smirnoff test.

MWU, Mann-Whitney U test.

FT, Fisher’s exact test.

**Table 1B:** Clinical characteristics of all participants in the study.

Metric	Patients with PMDD (N = 25)	Healthy Controls (N = 20)
Symptoms (years)	10.1 ± 6.9	–
Menarche age (years)	12.8 ± 1.7	12.3 ± 1.4
Oral contraception (y/n)	12/13	9/11
Physical training (y/n)	12/13	11/9
BMI	22.6 ± 4.1	22.9 ± 2.7
Smoking status (y/n)	13/12	10/10
Current pharmacological treatment (except psychiatric) (y/n)	4/21	0/20
Psychiatric history		
Psychiatric diagnosis (y/n)	12/13	0/20
Psychiatric pharmacology in past (y/n)	11/14	3/17
Current psychiatric pharmacology (y/n)	4/21	0/20
Current psychological treatment (y/n)	14/11	0/20

**Table 1C:** Psychiatric evaluation of patients with PMDD.

PMTS-OR	*Depression*	*Anxiety*	*Lability*	*Anger*	*Total*
	3.1 ± 0.9	3.1 ± 1.1	3.1 ± 1.2	3.2 ± 0.6	32.0 ± 6.2
BFI	*Extraversion*	*Neuroticism*	*Agreeableness*	*Conscientiousness*	*Openness to Experience*
	26.5 ± 6.3	25.1 ± 5.1	34.8 ± 3.6	31.9 ± 4.3	37.9 ± 5.5
CGI-S	5.1 ± 0.5				

PMTS-OR, Premenstrual Tension Syndrome Observer Rating Scale. Score range: Depression, 0 to 4; Anxiety, 0 to 4; Lability, 0 to 4; Anger, 0 to 4; total, 0 to 40.

BFI, Big Five Inventory. Score range: Extraversion, 8 to 40; Neuroticism, 8 to 40; Agreeableness, 9 to 45; Consientiousness, 9 to 45; Openness to Experience, 10 to 50.

CGI-S, Clinical Global Impressions, Severity. Score range: 1 to 7.

### Procedure

The experimental protocol included two visits to TASUMC, scheduled no more than one month apart. The first visit, conducted during the second week of the luteal phase (two to three days before the onset of menstruation), involved clinical assessments and lasted approximately 1.5 hours. The second visit included a neuroimaging session, performed on day 22.8 ± 3.1 of the menstrual cycle in the patient group (with the earliest scan conducted on day 17) and on day 20.8 ± 3.7 in the healthy group (with the earliest scan conducted on day 15). The study was approved by the Ethics Committee for Human Studies at TASUMC (approval number TLV-0034-15), and all procedures adhered to relevant ethical regulations. Written informed consent was obtained from all participants prior to their involvement in the study.

#### Imaging procedure

Neuroimaging data were acquired at TASUMC using a Siemens 3T MAGNETOM Prisma Scanner equipped with a 20-channel head coil. The total scanning session lasted approximately 30 minutes. Functional whole-brain scans were performed using T2*-weighted gradient echo-planar imaging (EPI) pulse sequence in interleaved order (TR/TE = 2500/35 ms; flip angle = 90°, voxel size 2.3×2.3×3.0 mm, FOV = 220 × 220 mm; slice thickness = 3 mm, no gap, 38 nearly horizontal slices per volume). High-resolution structural scans included a T1-weighted magnetization-prepared rapid gradient echo (MPRAGE) sequence (TR/TE = 1860/2.74 ms, flip angle = 8°, voxel size 1.0×1.0×1.0 mm, FOV = 256 × 256 mm, slice thickness = 1 mm, 176 contiguous slices). The structural scans were used for cortical segmentation and surface reconstruction.

#### fMRI task

During the fMRI scan, participants performed a task adapted from a previous study (29). In brief, participants completed *four* 6-minute sessions (each as a separate scan run) of the interactive computer game (Java1.6, Oracle, Redwood-Shores, CA & Processing package, http://www.processing.org), starting with a 1-minute baseline condition (black screen). In each session, participants played the game aimed to maximize their earnings by strategically focusing on either catching coins or avoiding balls, using response-box buttons to respond. To motivate approach behavior, participants were informed at the beginning of the experiment, as part of the experimental manipulation, that they would receive the total sum collected during the game as payment. However, at the conclusion of the task, the manipulation was disclosed, and all participants received a fixed payment. This fixed amount was higher than any possible earnings in the game and matched the standard compensation for participation in an fMRI experiment at our center.

**Figure 1:**
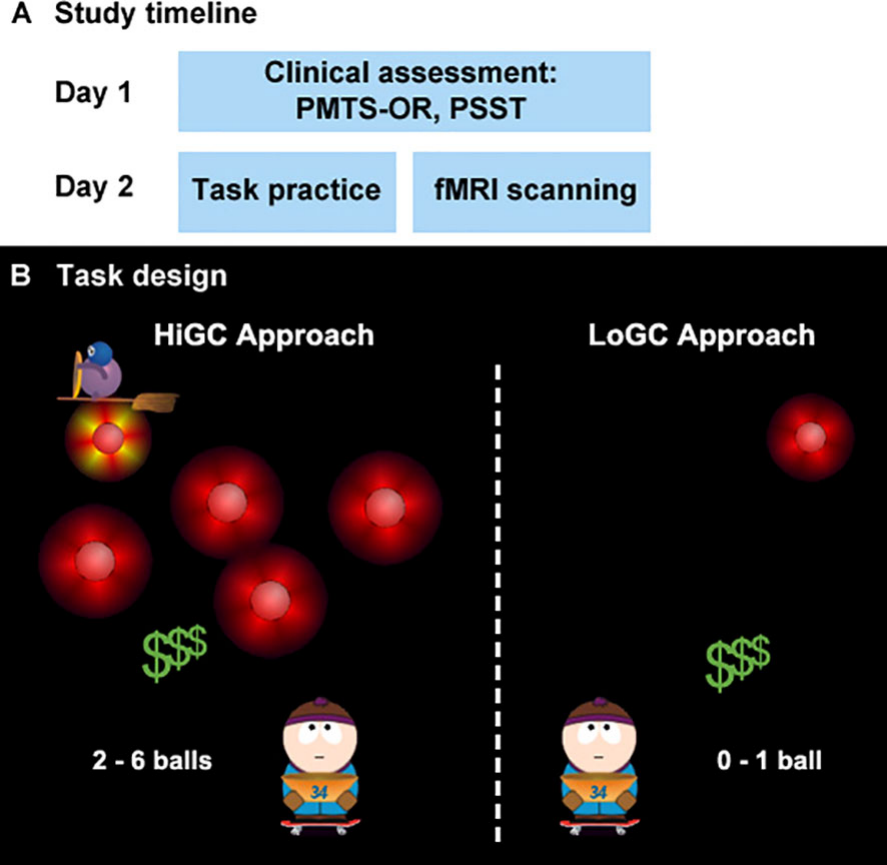
**(A)** Overview of the study design. The experimental protocol included two visits to TASUMC. The first visit, scheduled during the second week of the luteal phase, involved clinical assessments and lasted approximately 1.5 hours. The second visit included task practice (~1 minute) and an fMRI session (~ 30 minutes). **(B)** Schematic presentation of the task. Participants completed *four* 6-minute sessions of the interactive computer game, starting with a 1-minute black screen condition. In each session, participants played to maximize their earnings by adopting behavioral strategies inclined to either catching coins or avoiding balls. Monetary rewards were presented alongside potential punishments, creating either HiGC (2–6 balls between the player and coins) or LoGC (0–1 ball between the player and coins) goal conflict scenarios. The level of motivational conflict (i.e., number of balls) was dynamically adjusted every 10 seconds based on performance (success in catching coins). Each such trial within a session was isolated by an inter-stimulus interval (randomly varying between 550 and 2050 ms). Before the fMRI session, participants practiced the game for 1 minute outside the scanner. To encourage engagement, participants were informed that they would receive the total amount collected during the game.

In each session, two conditions were employed to earn or lose money: a ‘controlled’ trial, in which participants actively chose to approach the coins or avoid the balls, and an ‘uncontrolled’ trial, in which coins and balls were thrown at participants randomly. The number of balls between the player and the coins varied, creating either HiGC (2–6 balls between the player and coins) or LoGC (0–1 ball between the player and coins) approach conditions. The level of motivational conflict – defined as a number of balls – was dynamically adjusted every 10 seconds (i.e., per trial) based on performance (success in catching coins). Each trial within a session was isolated by an inter-stimulus interval (randomly varying between 550 and 2050 ms) to avoid overlap with the hemodynamic response function (36). At the end of each session, participants rated their feelings and level of attention toward each game condition using a 9-point Likert scale. Before the start of the fMRI session, participants practiced the game for one minute (five trials) outside the scanner to ensure familiarity with the task. **Figure 1** schematically illustrates the experimental paradigm.

### MRI image processing and data analysis

#### Preprocessing

fMRI data were analyzed using BrainVoyager QX (Brain Innovation, Maastricht, Netherlands), supplemented by in-house MATLAB (2020b) scripts. Analyses were first performed at the within-participant level (first-level analysis). Functional data preprocessing included head motion detection and correction using six rigid-body realignment parameters (three translation and three rotation parameters) with trilinear interpolation, followed by sinc interpolation for resampling. Temporal filtering was applied using a high-pass filter at 0.01 Hz with linear trend removal. Although a 6 mm kernel may be suboptimal for small structures such as the VTA, spatial specificity for subcortical ROIs was primarily ensured by the use of an independent functional localizer (see below), which constrains inference to empirically defined activation peaks rather than broad anatomical labels. The first six volumes from each run were excluded, to allow for signal stabilization. Structural images were normalized to Talairach space (37) and manually co-registered with the functional data. The pre-processed functional images were then integrated into 3D datasets using trilinear interpolation and projected onto reconstructed cortical surfaces generated from the anatomical images within BrainVoyager. Importantly, all statistical analyses were conducted in volume space, while cortical surface representations are shown for visualization purposes only.

Head motion was quantified by computing framewise displacement (FD; 38) from the six rigid-body realignment parameters estimated during motion correction algorithm. Rotational displacements were converted to millimeters assuming a nominal brain radius of 50 mm. Mean FD was averaged across runs for each participant. Group differences in mean FD and in the proportion of high-motion volumes (FD > 0.5 mm) were assessed using Mann–Whitney U tests. Mean FD did not differ between groups (Healthy: M = 0.171 ± 0.095 mm, median = 0.162 mm; PMDD: M = 0.198 ± 0.108 mm, median = 0.166 mm; U = 137.0, p = .442). The proportion of volumes exceeding FD > 0.5 mm was likewise comparable between groups (Healthy: 6.2 ± 6.6%; PMDD: 8.0 ± 8.5%; U = 135.0, p = .406). All participants remained below the commonly used data-quality threshold of mean FD < 0.5 mm.

#### Behavioral analyses

The labeling of game events as ‘approach’ (or ‘avoidance’) was derived from the game log files. A detailed description of the labeling algorithm is provided in (29). Briefly, the algorithm operated as follows: ten features were extracted from participants’ activity (e.g., response time, engagement, idle time, and keypad events), and a logistic regression model was trained based on these features using human-annotated event labels.

#### Selection of regions of interest

Regions of interest (ROIs) were defined using an independent localizer approach rather than anatomical atlases for two main reasons. First, several target structures, in particular the VTA and PAG, do not align well with standard atlas boundaries due to their small size and inter-individual anatomical variability, making atlas-based masks prone to including adjacent, functionally distinct tissue. Second, defining ROIs based on an independent, published HiGC > LoGC contrast from a separate healthy control sample (29) avoids circularity with respect to the present data. Spherical ROIs with a 3 mm radius were centered on subcortical activation peaks, while 5 mm spheres were used for cortical peaks. To ensure spatial specificity, each ROI center was verified against the Talairach Daemon database (https://www.talairach.org/daemon.html) and the Automated Anatomical Labelling atlas 3 (AAL3; 39); all centers fell within the expected anatomical regions (e.g., VTA centers within the midbrain tegmentum). ROI coordinates are reported in **Supplementary Tables 1**, **2**. *Post hoc* comparisons for each ROI were conducted using independent-samples t-tests (nine tests in total: bilateral VS, bilateral VTA, periaqueductal gray (PAG), bilateral MFG, right IFG, and left insula). Statistical significance was assessed using a false-discovery rate (FDR) correction at q <.05 (Benjamini-Hochberg procedure; 40, 41), applied across all nine tests simultaneously. Signal quality within each ROI was evaluated at the individual-subject GLM level; no participants were excluded from any ROI analysis due to signal dropout in the brainstem or other target regions.

#### Statistical analyses

Behavioral data were first analyzed by quantifying the number of approach events in each group. As detailed in the Results section, avoidance events did not differ significantly between groups and were therefore not included as a primary dependent variable. Notably, previous findings (29) also indicated that the paradigm primarily elicits approach behavior. For each participant, the number of approach events was calculated per session and averaged across sessions, and these group means were compared using an independent t-test. Next, to examine differences in approach events under the HiGC and LoGC conditions, the data were evaluated using a 2 (Group: PMDD vs. Healthy) × 2 (Conflict Level: Low vs. High) mixed design, with goal conflict condition treated as a within-subject factor and group treated as a between-subject factor. The repeated-measures structure was accommodated by modeling subject identity as a fixed effect in an ordinary least squares framework, which is equivalent to the corresponding repeated-measures ANOVA for this design. A parallel linear mixed-effects model was also fit to confirm the result, using random intercepts for subjects and, where the model converged, random slopes for goal conflict condition. Effect sizes were reported as partial eta squared (ηp² = SS_effect/(SS_effect + SS_error)). Planned comparisons tested group differences separately within each goal conflict level and tested the within-group HiGC versus LoGC contrast with paired-samples tests. Holm correction was applied to control family-wise error (α = .05), and Cohen’s d was calculated for each comparison.

#### Neuroimaging data

First-level (within-participant) neuroimaging data were analyzed using a general linear model (GLM) implemented in BrainVoyager QX, which is mathematically equivalent to standard GLM approaches used in SPM and FSL. Each participant’s BOLD time series was modeled separately (single-subject GLM), producing participant-level beta maps for each regressor of interest. Each game condition was defined as a distinct predictor (e.g., ‘approach HiGC’, ‘approach LoGC’). For each participant, these predictors were convolved with a standard hemodynamic response function (HRF). The global mean signal, the mean white-matter signal, the mean cerebrospinal fluid signal (CSF), extracted from a ventricle mask, and head-motion parameters were included as regressors in the GLM to account for non-neuronal physiological variance (38, 42). Subsequently, at the group level (second-level analysis), a whole-brain random-effect analysis (RFX-GLM) was performed (two-sample *t*-test, FDR-corrected).

Peak activation coordinates were reported in Talairach space, consistent with BrainVoyager’s output and the Talairach & Tournoux (37) atlas. To facilitate comparison with studies using MNI space, MNI equivalents for all peaks were computed using the Lancaster et al. (43) nonlinear Talairach-to-MNI transform (as implemented in NiMARE; 44) and are provided in **Supplementary Table 2**.

*Correlation with symptoms*: The numbers of approach events were correlated with PMDD symptoms (e.g., depression, anxiety, lability, and anger), as observed during a psychiatric examination, as well as core values. These correlations represent the relationship between motivational tendencies and symptom severity. Additionally, to examine whether neural activity across ROIs was associated with symptom severity, a multivariate repeated-measures model was fitted within the PMDD group (n = 20) using MATLAB^®^’s `fitrm` function. Beta values from the nine *a priori* ROIs (right and left VS, right and left VTA, PAG, right and left MFG, right and left IFG, and left insula) served as the within-subject (repeated) factor, with nine levels corresponding to the nine regions. Emotional symptom scores (depression, anxiety, lability, and anger), as assessed by the PMTS-OR, were entered as between-subject predictors. The significance of each predictor’s interaction with region was evaluated using `ranova` (degrees of freedom = 8, corresponding to nine regions − 1). For any symptom that reached significance in the test, region-specific associations were characterized in follow-up simple linear regressions (`fitlme`, fixed effects only), with each ROI’s beta value as the dependent variable and the significant symptom score as the sole predictor (residual degrees of freedom = 18, corresponding to n − 2). The false-positive rate across these follow-up tests was controlled at α = .05 without further correction, consistent with their exploratory character. Statistical analyses were performed using MATLAB^®^ routines from the Statistics Toolbox.

## Results

### Behavior under motivational goal conflict

To assess individual motivational behavior and differences between groups, approach and avoidance events were classified using a machine-learning-based algorithm (see Methods section). Consistent with previous findings (29), the paradigm primarily encouraged approach behavior, showing a marked bias toward approach events over avoidance.

To formally characterize this behavioral asymmetry, a 2 (Behavior: Approach vs. Avoidance) × 4 (Session) × 2 (Group: PMDD vs. Healthy) mixed-model ANOVA was conducted on the number of classified events per session. Results confirmed a highly significant main effect of Behavior [F(1, 315) = 4936.22, p <.001, η² = 0.940], with approach events (mean = 37.65 per session) substantially outnumbering avoidance events (mean = 5.15 per session) across both groups and all sessions. Critically, a separate analysis of avoidance events alone revealed no significant group difference [t(45) = −0.41, p = .682, d = −0.12], with avoidance counts increasing monotonically across sessions in both groups equally [Session effect: F(3, 135) = 19.90, p <.001, η² = 0.307; Session × Group: F(3, 135) = 0.53, p = .662], consistent with the adaptive difficulty algorithm increasing ball density as performance improved. The Behavior × Group interaction in the full ANOVA [F(1, 315) = 12.01, p = .001, η² = 0.037] reflected a modestly lower approach-to-avoidance ratio in the PMDD group (87%, range 84–90% across sessions) relative to the healthy group (89%, range 86–92% across sessions), driven entirely by group differences in approach events rather than avoidance. Because avoidance counts were not significantly different between groups and presumably primarily reflected task difficulty adaptation rather than motivational choice, subsequent analyses focused on approach events as the primary dependent variable.

**Figure 2:**
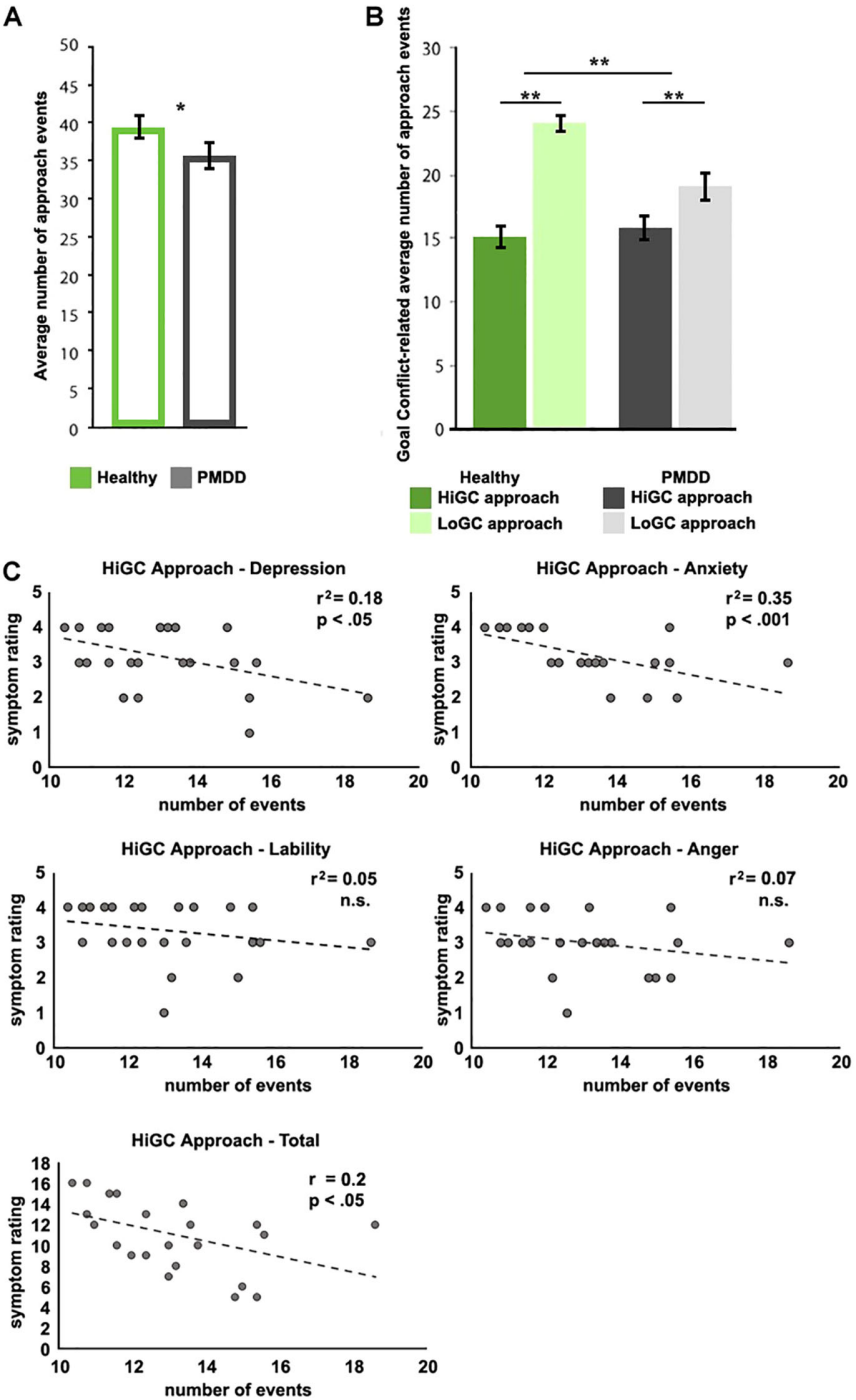
**(A)** Average number of approach events across all sessions. A significant group difference was observed, with the PMDD group exhibiting fewer approach events than the healthy group (independent *t*-test, p < .05). The proportion of approach events was 89% in the healthy group and 87% in the PMDD group (range: 86–92% and 84–90% across sessions, respectively), confirming the predominance of approach behavior in both groups. **(B)** *Effect of conflict level.* A 2 × 2 between-subjects ANOVA revealed significant main effects of conflict (p <.001) and group (p = .001). Approach events occurred significantly more frequent under LoGC than HiGC conditions, consistent with previous findings (29). The interaction reflected a markedly attenuated goal conflict-related increase in the PMDD group relative to healthy group (p <.001). Error bars represent SD in all graphs. **(C)** Association between symptom severity and approach behavior under HiGC conditions in the PMDD group. Depression and anxiety, as well as total PMTS-OR scores, were significantly negatively correlated with the number of approach events under HiGC conditions. No significant correlations were observed for lability or anger scores. Note that in some plots, the data points for a few participants (out of 25) overlap exactly on both dimensions, resulting in some circles being obscured and therefore not visible.

The average number of approach events across all game sessions was calculated for both groups (**Figure 2A**). As expected, the PMDD group exhibited significantly fewer approach events than the healthy group (independent *t*-test, *p* < .05). Variation in the number of approach events across sessions within both groups was minimal, likely reflecting real-time risk-level adjustments based on individual performance.

To examine the effect of conflict level, as defined by the number of risk-inducing obstacles, approach events under HiGC and LoGC conditions were analyzed using a 2 (Group: PMDD vs. Healthy) × 2 (Conflict Level: Low vs. High) mixed-design analysis with conflict level specified as a within-subject factor (**Figure 2B**). The repeated-measures structure was accommodated by modeling subject identity as a fixed effect in an ordinary least squares framework, with a parallel linear mixed-effects model providing a confirmatory check. Results showed a strong main effect of Conflict (F(1, 43) = 122.80, p <.001, ηp² = 0.74) and a significant Group × Conflict interaction (F(1, 43) = 36.92, p <.001, ηp² = 0.46), while the main effect of group was not significant (F(1, 43) = 0.83, p = .369). Means and standard deviations were: Healthy – HiGC: 15.19 (2.09), LoGC: 23.73 (2.93); PMDD – HiGC: 16.37 (2.40), LoGC: 19.06 (2.52). The Conflict effect (Low > High) was larger in the healthy group (Δ = 8.54) than in the PMDD group (Δ = 2.70). *Post-hoc* tests (Holm-adjusted p-values) showed Group at HiGC nonsignificant, t(43) = -1.73, p = .091, d = -0.52; Group at LoGC: Healthy > PMDD, t(43) = 5.73, p <.001, d = 1.72; Conflict within healthy group: Low > High, t(19) = -12.83, p <.001, d = -2.87; Conflict within PMDD group: Low > High, t(24) = -3.996, p = .0011, d = -0.80. The interaction reflected a markedly attenuated goal conflict-related increase in approach behavior in the PMDD group relative to the healthy group, suggesting that PMDD participants showed a reduced motivational sensitivity to increasing goal conflict compared with healthy controls.

Next, we examined whether approach behavior under HiGC conditions correlated with symptom severity in patients with PMDD (**Figure 2C**). As expected, higher levels of depression and anxiety symptoms, as well as higher total PMTS-OR scores, were associated with reduced approach behavior under HiGC conditions, showing significant negative correlations (*p* < .05). However, no significant correlations were found for emotional lability or anger scores.

### Brain activation under motivational goal conflict

**Figure 3:**
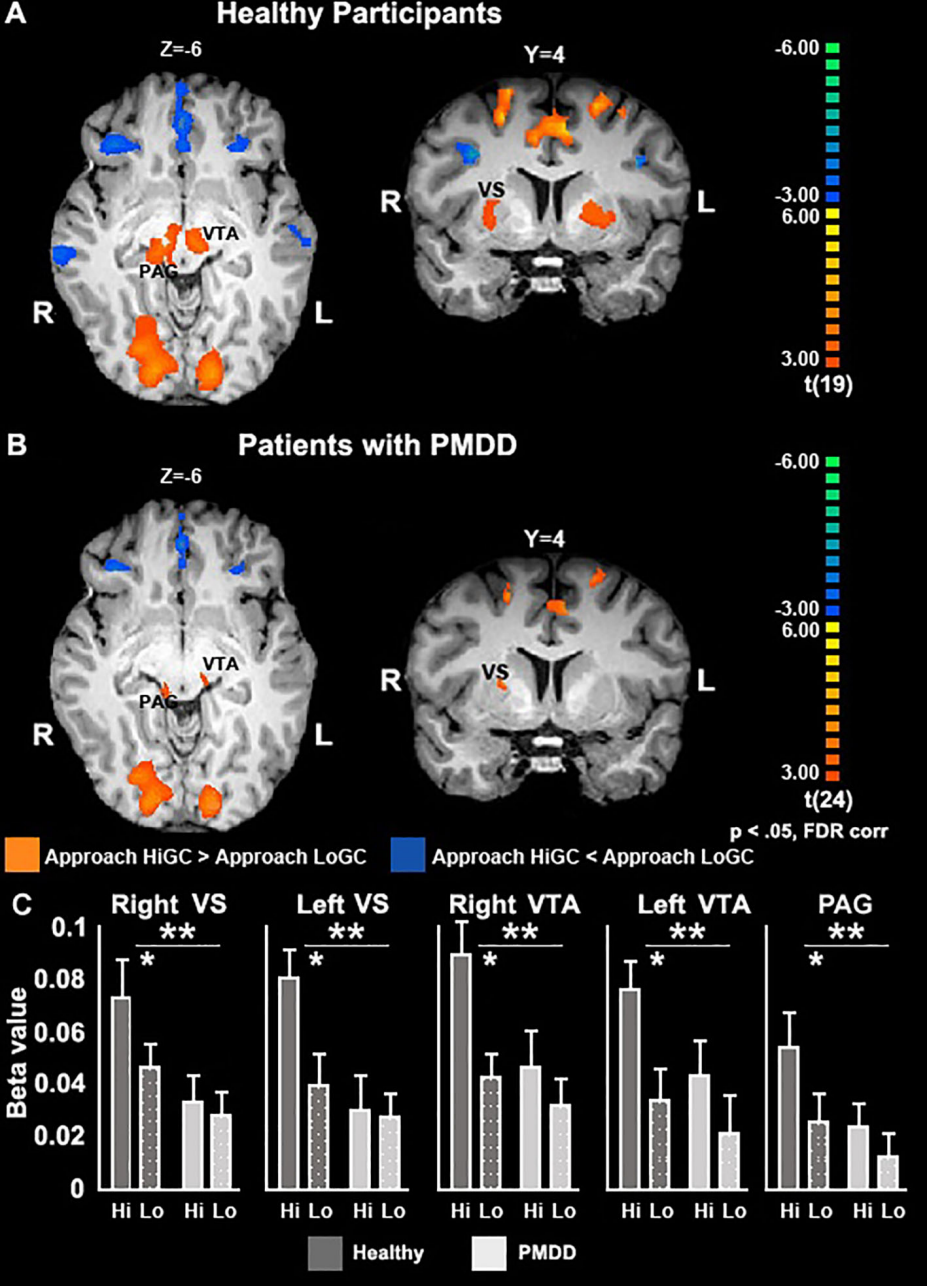
Whole-brain analysis: subcortical regions. **(A)** In the healthy group, significantly higher activation was observed under HiGC vs. LoGC conditions in the VTA and VS, with increased activation also noted in the PAG. **(B)** In the PMDD group, subcortical regions exhibited weaker and less consistent activation, with a noticeable reduction compared to the healthy controls. **(C)** Quantitative analysis. Two-tailed *t*-tests were conducted on beta values derived from the independent ROIs identified in the HiGC vs. LoGC contrast in the additional data set of healthy controls. The bilateral VS, bilateral VTA, and PAG showed significantly higher beta values under HiGC condition in healthy group. Asterisks represent *post hoc* comparisons after FDR correction for multiple comparisons; *p < .05, **p < .01.

We first conducted a whole-brain analysis to examine neural differences in motivational goal conflict within each group. Comparing activity under the HiGC and LoGC approach conditions revealed that the healthy group showed significantly greater activation in the VTA and VS (p < .05, FDR-corrected; **Figure 3A**), consistent with our hypotheses. Increased activation was also observed in the PAG, a region implicated in motivation and threat responses. In contrast, the PMDD group exhibited weaker and less consistent activation in these regions, with a marked reduction in neural responses compared to healthy controls (**Figure 3B**).

To quantify the observed differences and compare neural activity between groups, beta values were extracted from ROIs defined using the same HiGC vs. LoGC contrast in a cohort of healthy participants (see Methods, **Figure 3C**). *Post hoc* comparisons using *t*-tests were then applied to these beta values, with significance assessed after correction for multiple comparisons. As predicted, the healthy group showed significantly higher (p < .01) beta values under the HiGC condition in the bilateral VS [Talairach coordinates: left (-16, 1, 3), right (23, 7, 0)], bilateral VTA [left (-13, -14, -6), right (11, -17, -3)], and in the PAG (-4, -29, -3).

**Figure 4:**
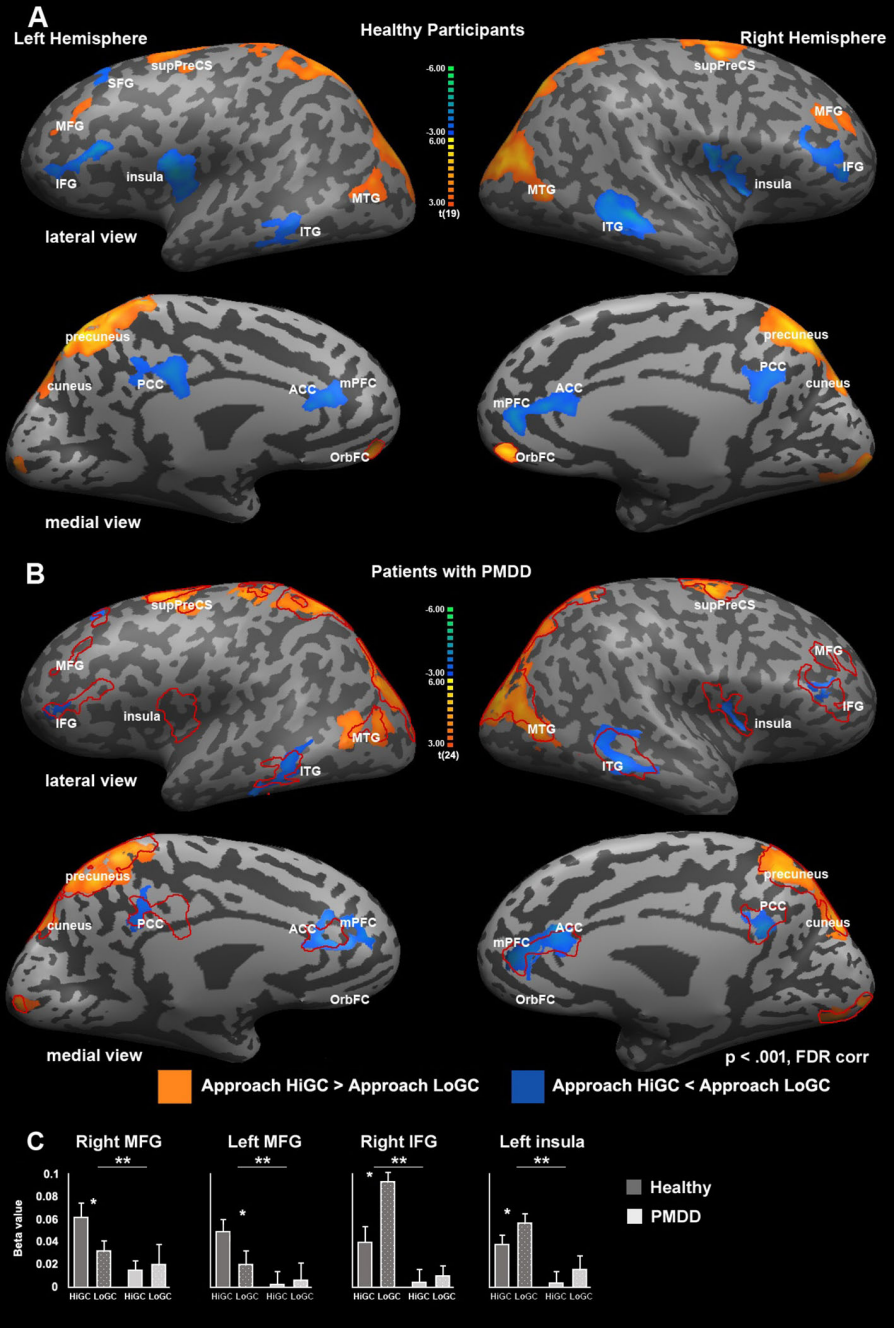
Whole-brain analysis: cortical regions. **(A)** In the healthy group, HiGC conditions elicited increased activity bilaterally in the superior precentral gyri, medial frontal gyrus (MFG), middle temporal gyri (MTG), inferior occipital gyri (IOG), orbitofrontal cortex (OrbFC), precuneus, and cuneus (orange clusters, p < .001, FDR-corrected). **(B)** In the PMDD group, a similar activation pattern was observed under HiGC conditions, except for the lack of bilateral MFG activity. Under LoGC conditions, both groups showed increased activation in the inferior temporal gyri (ITG), inferior frontal gyri (IFG), posterior insula, anterior and posterior cingulate, and medial prefrontal cortex (mPFC), including the right superior frontal gyrus (SFG) (blue clusters). The red outline indicates overlapping activation patterns between the two groups. **(C)** Quantitative analysis. The bilateral MFG showed significantly higher beta values in HiGC condition in healthy group. In contrast, the right IFG and left insula showed significant preference for the LoGC. Asterisks represent *post hoc* comparisons after FDR correction for multiple comparisons; *p < .05, **p < .01.

A broad set of cortical regions also showed significantly greater activation under HiGC compared to LoGC conditions (p < .001, FDR-corrected; **Figure 4**). In the healthy group, this included bilateral superior precentral gyri (premotor cortices), MFG, middle temporal gyri (MTG), inferior occipital gyri (IOG, visual areas BA 17/18), orbitofrontal cortex (OrbFC) and extensive regions of the precuneus and cuneus (**Figures 4A**, orange). The PMDD group showed a generally similar pattern under HiGC, but lacked the preferential activation observed in the bilateral MFG (**Figures 4B**, orange). Under LoGC conditions, both groups showed increased activation in the inferior temporal gyri (ITG), IFG, posterior insula, anterior and posterior cingulate, and mPFC, including the right superior frontal gyrus (SFG). However, the left posterior insula was not activated in the PMDD group (**Figures 4A, B**, blue). The red outline in **Figure 4B** highlights overlapping activation maps between groups, showing more extensive activity in the healthy group across all aforementioned regions. The quantitative analyses revealed significantly higher beta values under the HiGC condition in the bilateral MFG (left [-31, 35, 35]; right [29, 34, 36]), whereas the right IFG [38, 16, 24] and left insula [-40, 5, 0] showed preferential activity to LoGC (**Figure 4C**).

The effect sizes of the group comparisons ranged from medium to large: left VS – 0.7, right VS – 0.7, left VTA – 0.75, right VTA – 0.75, PAG – 0.58, right MFG – 0.68, left MFG – 0.65, right IFG – 0.51, left insula – 0.48. Additional details about obtained activations (i.e., Talairach/MNI coordinates for activation peaks, cluster size, etc.) are provided in **Supplementary Tables 1**, **2**.

### Symptom severity and neural activity

We further investigated whether neural activity across the *a priori* ROIs was associated with symptom severity within the PMDD group. A multivariate repeated-measures model was fitted with beta values from the nine ROIs as the within-subject factor and the four emotional symptom scores (depression, anxiety, lability, and anger) as between-subject predictors. The analysis indicated that depression was the only symptom with a significant interaction with region [F(8, 11) = 2.10, p = .04], reflecting a differential association between depression severity and neural activity across the nine ROIs. The corresponding effects for anxiety [F(8, 11) = 1.24, p = .28], lability [F(8, 11) = 0.69, p = .70], and anger [F(8, 11) = 0.91, p = .51] were not significant.

**Figure 5:**
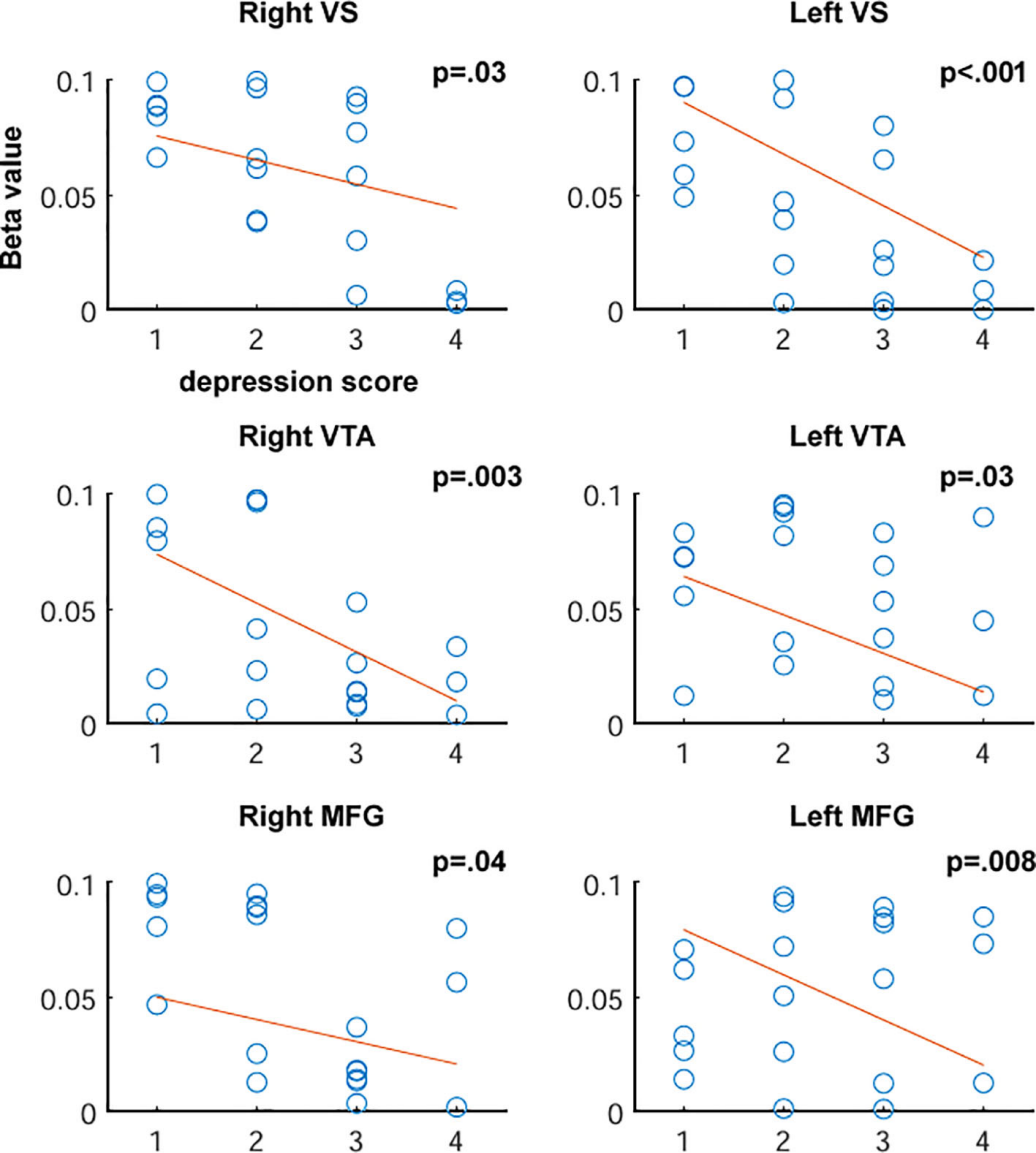
Association between symptom severity and neural activity. A multivariate repeated-measures model was conducted with beta values from the nine ROIs as the within-subject factor and the four emotional symptom scores (depression, anxiety, lability, and anger) as between-subject predictors. Depression emerged as the only significant predictor of neural activity. Depression severity significantly predicted beta values in six regions: VS, VTA, and MFG bilaterally. In all six regions, higher depression scores were associated with lower beta values under the HiGC condition.

Given the significant effect of depression, follow-up region-specific regressions were conducted to identify which ROIs drove this association (**Figure 5**). Depression severity significantly predicted beta values in six of the nine regions: right VS (t(18) = −2.40, p = .03), left VS (t(18) = −4.91, p <.001), right VTA (t(18) = −3.50, p = .003), left VTA (t(18) = −2.41, p = .027), right MFG [t(18) = −2.19, p = .041], and left MFG (t(18) = −2.98, p = .008). In all six regions, higher depression scores were associated with lower beta values under the HiGC condition, suggesting that greater depressive symptom burden is linked to attenuated activation of reward-related and prefrontal motivational circuitry.

## Discussion

To our knowledge, few if any studies have directly examined motivational conflict behavior using a naturalistic gamified fMRI paradigm in individuals with PMDD. By employing a naturalistic, gamified context of goal conflict in combination with fMRI, we demonstrated that motivational behavior can be modulated within goal-conflict situations (defined as HiGC or LoGC). While our findings align with previous results in healthy individuals (29), they also highlight significant behavioral and neural deviations in individuals with PMDD. These findings provide novel insights into the neurobehavioral correlates of motivational deficits in PMDD, with potential implications for future research on diagnostic subtyping and intervention targets.

### Motivational behavior deficit in PMDD

Our findings indicated that individuals with PMDD exhibit impaired motivational adaptation across goal-conflict levels, rather than a uniform reduction in approach behavior. Although both groups showed more approach events under LoGC than under HiGC conditions, this conflict-related increase was markedly attenuated in the PMDD group compared with healthy controls (interaction p <.001; **Figure 2B**). The significant between-group difference was confined to the LoGC condition, in which healthy participants exhibited substantially more approach events than individuals with PMDD (p <.001), whereas no significant group differences were observed under HiGC (p = .091; **Figure 2A**). These results suggest that individuals with PMDD have a reduced ability to scale up approach behavior when motivational conflict is low – that is, when pursuing rewards is relatively less risky – indicating an impaired capacity to flexibly adapt goal-directed behavior in response to changing levels of risk and opportunity.

These results align with reinforcement theory (45), which posits that the behavioral approach system drives reward-seeking behavior. Core symptoms of PMDD during the luteal phase, including reduced motivation, loss of interest in usual activities, fatigue and low energy may reflect disruptions in this system (46). Prior research has demonstrated that luteal-phase hormonal fluctuations, particularly involving estrogen and progesterone, can influence reward-related neural circuitry (47, 48). Our findings are consistent with this broader literature – participants were scanned during the luteal phase, when PMDD symptoms peak – and extend it by showing that impaired reward responsiveness manifests in goal-directed behavior under conditions of motivational conflict. In clinically intuitive terms, this pattern suggests that individuals with PMDD may fail to fully increase engagement in rewarding actions even when situational risk is reduced. This pattern aligns with prior reports of reduced reward sensitivity and diminished behavioral adaptation to reinforcement cues in women with premenstrual syndrome during the luteal phase (46, 49). However, because the present study did not include concurrent hormone assays, a direct causal link between hormonal fluctuations and the observed behavioral and neural differences cannot be established. Future studies incorporating concurrent hormonal measurements and more precise control of scan timing will be necessary to directly evaluate hormonal mechanisms.

Altogether, these results suggest that hormonal shifts may influence mesolimbic reward circuitry, potentially contributing to the motivational and emotional difficulties observed in PMDD. Whether this modulation specifically involves abnormal dopaminergic mechanisms remains to be determined in studies employing direct measures of dopamine release or receptor function.

### Neural mechanisms of motivational deficits in PMDD

The blunted conflict-related modulation of approach behavior in the PMDD group was mirrored by diminished activity in key mesostriatal reward-related regions under HiGC conditions. In the healthy group, activity in the VTA and VS increased as a function of goal conflict, consistent with the role of these regions in reward processing and motivational behavior. In contrast, individuals with PMDD showed attenuated and inconsistent activation in these regions during HiGC conditions (**Figure 3C**). Our findings align with established models of reward processing, which emphasize the role of mesolimbic circuitry in regulating approach behavior under conditions of risk (50), and are consistent with — though do not directly test — reinforcement-learning frameworks (e.g., reward prediction error models) that posit a central role for dopaminergic signaling in the mesolimbic system. The lack of conflict-dependent modulation in the PMDD group suggests an impaired ability to engage these neural circuits when motivational behavior requires balancing reward and risk – a critical component of effective coping with stress (51).

Finally, we found that depression was the only significant variable accounting for variance in activation differences in key mesostriatal regions (i.e., the VS and VTA), which are critical for reward processing and motivation, as well as in the prefrontal region (i.e., MFG), implicated in motivational value-based decision making (52). This result suggests that variability in depression severity is associated with differences in motivation-related brain activity in individuals with PMDD. Indeed, anhedonia, a core and often persistent symptom of depression (even following successful mood stabilization) has been consistently linked to dysfunction within the mesolimbic circuit, particularly involving the NA and VTA (53–57). These findings highlight a potential therapeutic direction for addressing depressive symptoms in PMDD, particularly from a behavioral perspective focused on motivation and goal-directed behavior. Moreover, our results in the MFG align with recent neuroimaging studies showing that women with PMDD exhibit hypoactivation in brain regions involved in emotion regulation, particularly the right dorsolateral prefrontal cortex, during the luteal phase (58). Such neural hypoactivation correlates with increased negative affect and impaired emotion regulation, further underscoring the impact of depressive symptoms on altered brain function in PMDD.

### Limitations and future directions

The study has several limitations. First, it did not assess whether responses to reward consumption itself were impaired. Future research should explore this issue to clarify whether PMDD-related deficits extend to reward anticipation versus reward consumption. Second, the relatively small sample size may have limited the statistical power of some analyses. Additionally, some patients in the PMDD group received pharmacological (e.g., fluoxetine, escitalopram, sertraline) and/or psychological treatments, which could have influenced the results. We did not explore these questions in depth due to the limited number of participants who received the treatment. However, analyses conducted in clinically homogeneous subgroups revealed no significant differences (see **Supplementary Materials**). These included: (i) patients not currently receiving pharmacological treatment (N = 21; **Supplementary Document 1**); (ii) patients *with* (N = 12) and *without* (N = 13) a prior history of psychopharmacological treatment (**Supplementary Document 2**); and (iii) patients *with* (N = 14) and *without* (N = 11) psychological treatment (**Supplementary Document 3**). These findings suggest that such factors are unlikely to have influenced the results. Nonetheless, studies with larger samples are needed to confirm these findings. Third, because the study was conducted during the luteal phase, the findings may not generalize to other phases of the menstrual cycle. Moreover, interpreting correlations between behavioral/fMRI results and PMDD symptom severity may be complicated by hormonal mismatch. Although both symptom severity and fMRI scanning were assessed during the luteal phase, they were not conducted on the same date. Symptom severity was evaluated toward the late luteal phase, whereas scanning occurred earlier. Given that estrogen and progesterone levels peak during the mid-luteal phase and estrogen declines toward the late luteal phase, more precise temporal alignment of these assessments would be preferable. Nevertheless, as PMDD symptoms peak during the luteal phase, this window remains particularly relevant for investigating the associated motivational and neural impairments. In the absence of concurrent hormone assays, any discussion of estrogenic or progestogenic mechanisms in this work should be considered strictly hypothesis-generating rather than conclusive.

In conclusion, this study provides novel insights into the neural and behavioral underpinnings of motivational deficits in individuals with PMDD, demonstrating that the characteristic emotional difficulties of this condition extend to goal-directed behavior under conditions of motivational conflict. The VS and VTA – key nodes of mesostriatal reward circuitry – showed conflict-dependent hypoactivation in the PMDD group, and this neural pattern was specifically associated with depression severity. Notably, similar alterations in mesolimbic reward circuitry and motivation have been consistently reported in major depressive disorder, suggesting a potential shared mechanism across conditions, although this comparison was not directly tested in the present study (57). Future research should examine whether these motivational and neural alterations translate into real-world difficulties in goal-pursuit, and whether targeted interventions that engage reward-processing pathways can enhance motivational flexibility in individuals with PMDD and related mood disorders.

## Data Availability

The raw data supporting the conclusions of this article will be made available by the authors, without undue reservation.
