# Supplementary material for: Motivational neurobehavioral abnormalities under a naturalistic goal-conflict task in patients with premenstrual dysphoric disorder
Source: Front Psychiatry. 2026 Jun 8;17:1776826. doi: 10.3389/fpsyt.2026.1776826 (PMC13285178; doi:10.3389/fpsyt.2026.1776826)
Supplement: Supplementary file 1 [file Supplementaryfile1.docx]

***Results for a subgroup of patients currently not receiving pharmacological treatment***

1. **Clinical characteristics**

| **PMTS-OR** | *Depression* | *Anxiety* | *Lability* | *Anger* | *Total* |
| --- | --- | --- | --- | --- | --- |
|  | 3.3 ± 0.7 | 3.2 ± 0.7 | 3.3 ± 0.8 | 3.0 ± 0.7 | 31.6 ± 4.5 |

| **BFI** | *Extraversion* | *Neuroticism* | *Agreeableness* | *Conscientiousness* | *Openness to experience* |
| --- | --- | --- | --- | --- | --- |
|  | 25.9 ± 5.5 | 26.1 ± 5.5 | 34.5 ± 4.1 | 31.4 ± 4.2 | 38.5 ± 6.2 |

| **CGI-S** | 5.2 ± 0.6 |
| --- | --- |

[PMTS-OR] Premenstrual Tension Syndrome Observer Rating Scale. Score range: Depression, 0 to 4; Anxiety, 0 to 4; Lability, 0 to 4; Anger, 0 to 4; Total, 0 to 40.

[BFI] Big Five Inventory. Score range: Extraversion, 8 to 40; Neuroticism, 8 to 40; Agreeableness, 9 to 45; Consientiousness, 9 to 45; Openness to Experience, 10 to 50.

[CGI-S] Clinical Global Impressions, Severity. Score range: 1 to7.

1. **Behavioral comparison with the initial PMDD group**

**
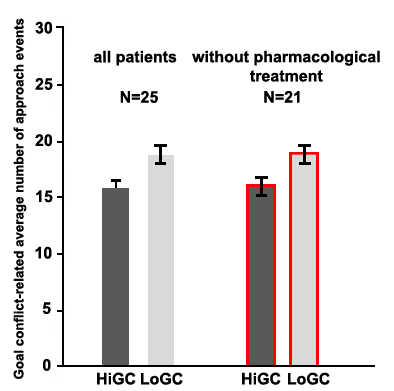
Figure S1.** We compared the average number of approach events across all sessions revealed in the whole PMDD group (N = 25) and in a subgroup of patients that did not receive pharmacological treatment (N =21). The findings are very similar to the outcomes found initially.

1. **Behavior-symptom association**

**Figure S2**. Analyses of association between symptom severity and approach behavior under HiGC conditions were repeated for the patient subgroup without current pharmacological treatment. Namely, depression and anxiety, as well as total PMTS-OR scores, were significantly negatively correlated with the number of approach events under HiGC conditions. No significant correlations were observed for lability or anger scores. Note that in some plots, the data points for a few participants overlap exactly on both dimensions, resulting in some circles being obscured and therefore not visible.

**
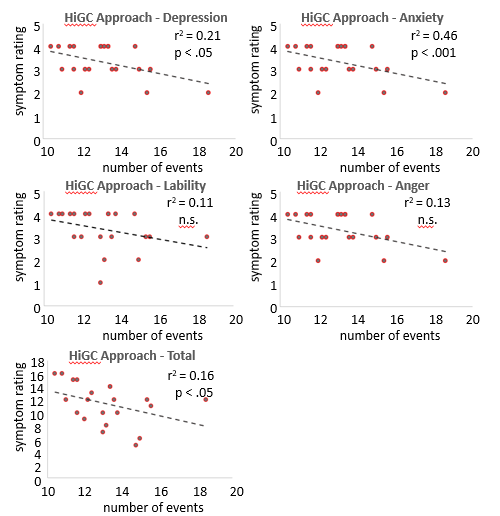
**

1. **Neural comparison**


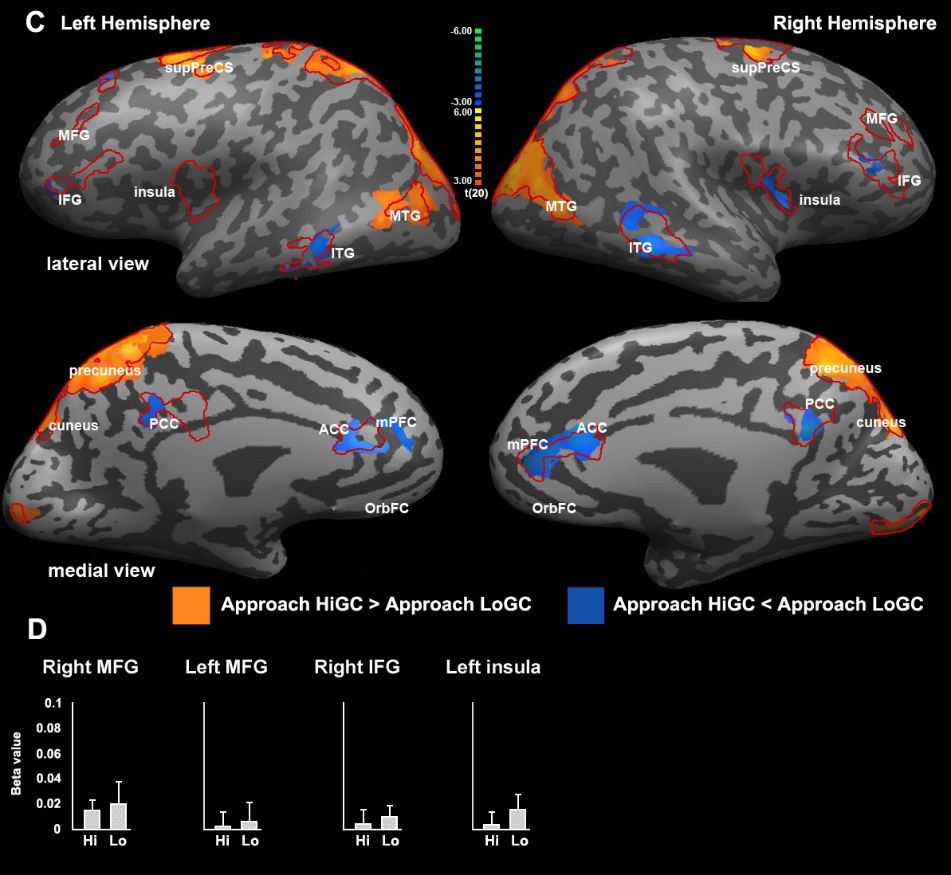

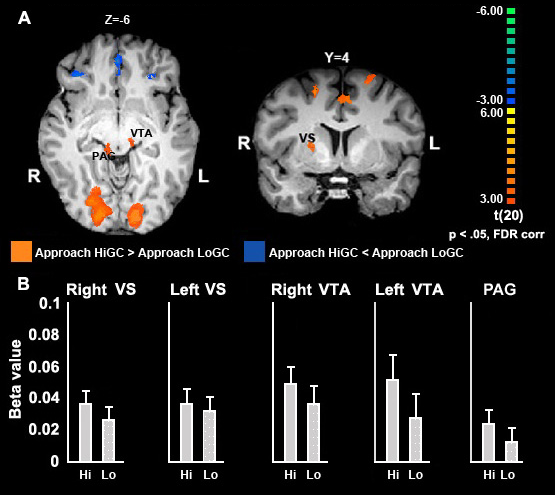
**Figure S3**. Whole-brain (A & C) and quantitative (B & D) analyses were repeated on 21 patients who completed the fMRI task and did not receive psychiatric medication for their PMDD symptoms. The resulting maps were highly similar to those obtained for the entire PMDD group (see Figure 3 in the main text). In addition, a direct comparison between the two sets of data did not reveal any significant differences.
