# Supplementary material for: Motivational neurobehavioral abnormalities under a naturalistic goal-conflict task in patients with premenstrual dysphoric disorder
Source: Front Psychiatry. 2026 Jun 8;17:1776826. doi: 10.3389/fpsyt.2026.1776826 (PMC13285178; doi:10.3389/fpsyt.2026.1776826)
Supplement: Supplementary file 2 [file Supplementaryfile2.docx]

***Comparison between patients with (N=12) and without (N=13) prior psychopharmacological treatment***

1. **Clinical characteristics**

| **PMTS-OR** |  | *Depression* | *Anxiety* | *Lability* | *Anger* | *Total* |
| --- | --- | --- | --- | --- | --- | --- |
|  | ***with*** | 3.2 ± 0.8 | 3.3 ± 0.6 | 3.2 ± 1.0 | 2.6 ± 0.8 | 30.4 ± 4.3 |
|  | ***without*** | 3.3 ± 1.1 | 3.3 ± 0.7 | 3.4 ± 0.9 | 3.0 ± 0.7 | 32.9 ± 4.6 |

| **BFI** |  | *Extraversion* | *Neuroticism* | *Agreeableness* | *Conscientiousness* | *Openness to experience* |
| --- | --- | --- | --- | --- | --- | --- |
|  | ***with*** | 27.8 ± 6.1 | 24.8 ± 6.1 | 34.3 ± 4.6 | 31.4 ± 4.5 | 39.4 ± 7.0 |
|  | ***without*** | 26.0 ± 6.5 | 26.7 ± 4.9 | 34.6 ± 3.6 | 32.0 ± 4.2 | 38.3 ± 5.6 |

| **CGI-S** | ***with*** | 5.16 ± 0.41 |
| --- | --- | --- |
|  | ***without*** | 5.11 ± 0.60 |

[PMTS-OR] Premenstrual Tension Syndrome Observer Rating Scale. Score range: Depression, 0 to 4; Anxiety, 0 to 4; Lability, 0 to 4; Anger, 0 to 4; Total, 0 to 40.

[BFI] Big Five Inventory. Score range: Extraversion, 8 to 40; Neuroticism, 8 to 40; Agreeableness, 9 to 45; Consientiousness, 9 to 45; Openness to Experience, 10 to 50.

[CGI-S] Clinical Global Impressions, Severity. Score range: 1 to7.

No significant differences were found between patients with and without previous psychiatric treatment. However, a trend was observed in some scores (e.g., lability, anger, and total), with slightly higher values among patients without a prior history of psychopharmacological treatment.

1. **Behavioral comparison between subgroups**

**
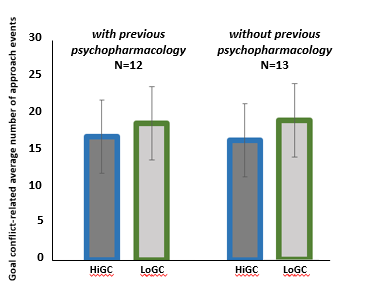
Figure S4.** The average number of approach events across all sessions was compared between patients with and without a prior history of psychopharmacological treatment. No significant between-group differences were observed, and the findings were consistent with those obtained in the full sample.

1. **Neural comparison**


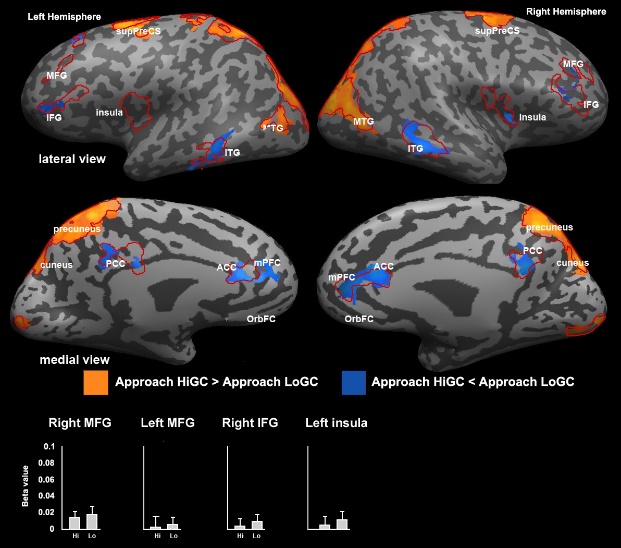

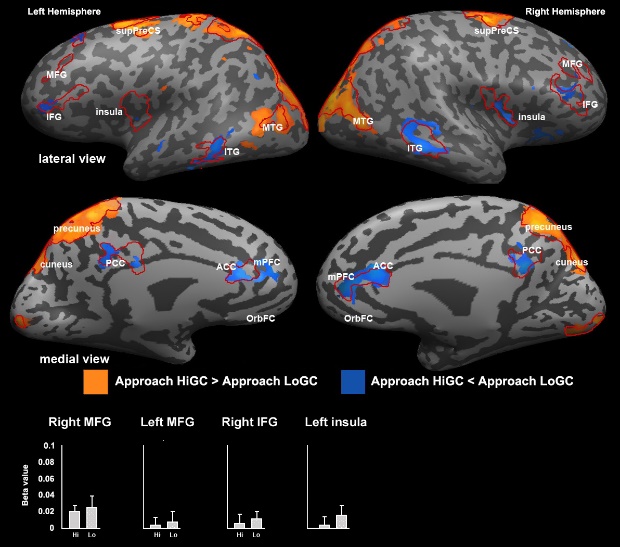

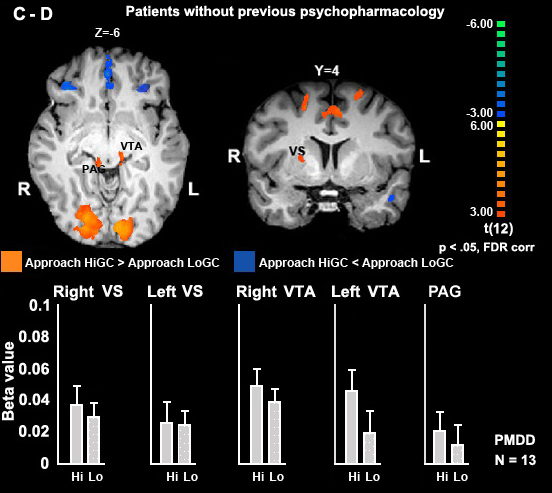

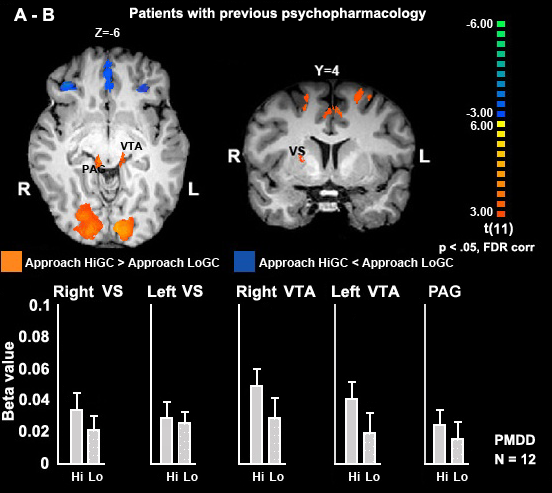
**Figure S5**. Whole-brain and quantitative analyses were repeated in 12 patients with a history of psychiatric medication for their conditions and in 13 patients without such treatment. The results were similar across groups, although the maps were slightly noisier than those obtained for the full sample. No significant differences were observed between the two groups.
