# Supplementary material for: Motivational neurobehavioral abnormalities under a naturalistic goal-conflict task in patients with premenstrual dysphoric disorder
Source: Front Psychiatry. 2026 Jun 8;17:1776826. doi: 10.3389/fpsyt.2026.1776826 (PMC13285178; doi:10.3389/fpsyt.2026.1776826)

***Comparison between patients with (N=14) and without (N=11) psychological treatment***

1. **Clinical characteristics**

| **PMTS-OR** |  | *Depression* | *Anxiety* | *Lability* | *Anger* | *Total* |
| --- | --- | --- | --- | --- | --- | --- |
|  | ***with*** | 3.1 ± 0.7 | 3.1 ± 0.7 | 3.3 ± 0.7 | 2.7 ± 0.7 | 30.4 ± 4.9 |
|  | ***without*** | 3.2 ± 1.0 | 3.3 ± 0.6 | 3.3 ± 0.9 | 3.3 ± 0.5 | 32.4 ± 4.3 |

| **BFI** |  | *Extraversion* | *Neuroticism* | *Agreeableness* | *Conscientiousness* | *Openness to experience* |
| --- | --- | --- | --- | --- | --- | --- |
|  | ***with*** | 27.7 ± 6.5 | 23.7 ± 5.8 | 34.6 ± 4.4 | 30.7 ± 4.0 | 39.2 ± 6.4 |
|  | ***without*** | 26.2 ± 6.1 | 27.4 ± 4.8 | 34.3 ± 3.9 | 32.5 ± 4.5 | 38.5 ± 6.3 |

| **CGI-S** | ***with*** | 5.0 ± 0.0 |
| --- | --- | --- |
|  | ***without*** | 5.15 ± 0.55 |

[PMTS-OR] Premenstrual Tension Syndrome Observer Rating Scale. Score range: Depression, 0 to 4; Anxiety, 0 to 4; Lability, 0 to 4; Anger, 0 to 4; Total, 0 to 40.

[BFI] Big Five Inventory. Score range: Extraversion, 8 to 40; Neuroticism, 8 to 40; Agreeableness, 9 to 45; Consientiousness, 9 to 45; Openness to Experience, 10 to 50.

[CGI-S] Clinical Global Impressions, Severity. Score range: 1 to7.

No significant differences were found between patients receiving and not receiving psychological treatment. However, a trend was observed in several scores (e.g., anger, total, neuroticism, and conscientiousness), with slightly higher values among patients not receiving psychological treatment.

1. **Behavioral comparison between subgroups**

**
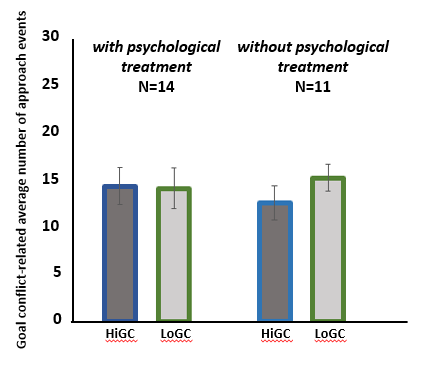
Figure S6.** The average number of approach events across all sessions was compared between patients receiving and not receiving psychological treatment. No significant between-group differences were observed, and the findings were consistent with those obtained in the full sample.

1. **Neural comparison**


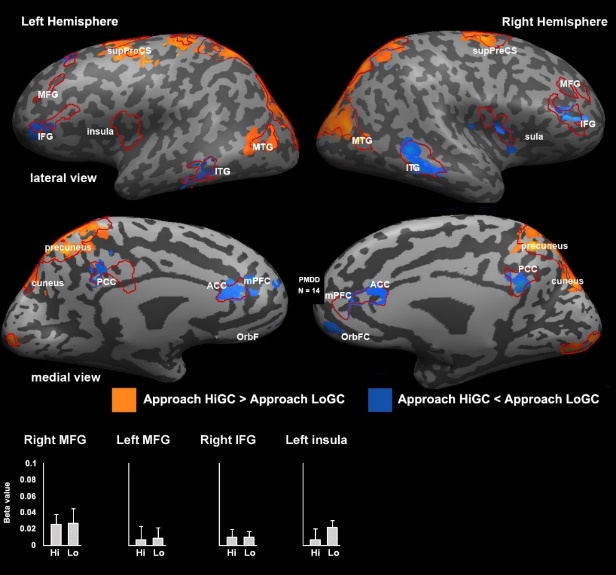

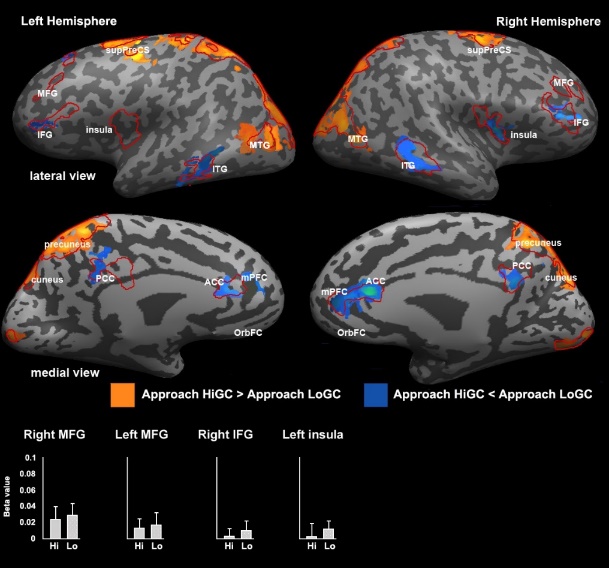

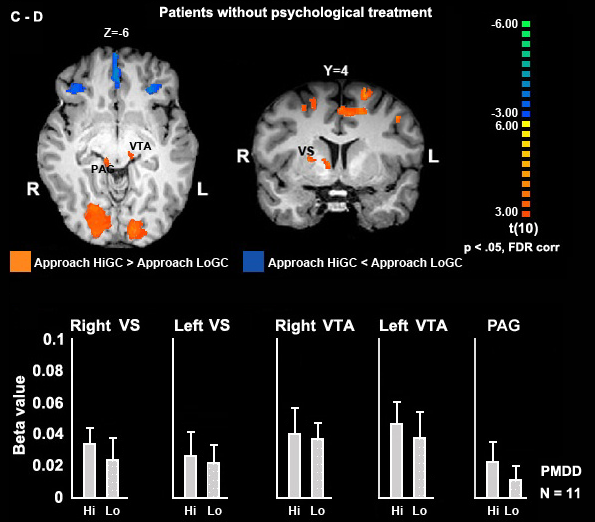
**Figure S7**. Whole-brain and quantitative analyses were repeated in 14 patients receiving psychological treatment and 11 patients without such treatment. No significant differences were observed between the two subgroups, although the maps were noisier than in the full sample.


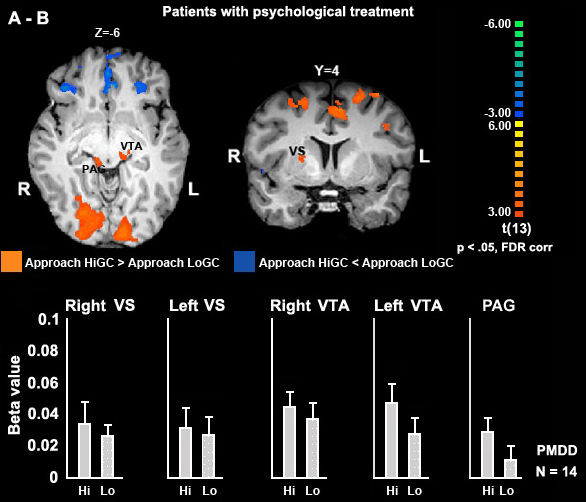

Supplement: Supplementary file 3 [file Supplementaryfile3.docx]
