# Supplementary material for: Motivational neurobehavioral abnormalities under a naturalistic goal-conflict task in patients with premenstrual dysphoric disorder
Source: Front Psychiatry. 2026 Jun 8;17:1776826. doi: 10.3389/fpsyt.2026.1776826 (PMC13285178; doi:10.3389/fpsyt.2026.1776826)
Supplement: Supplementary file 4 [file Supplementaryfile4.docx]

**Table S2****.** **Brain regions showing significant activation differences (HiGC vs LoGC approach), in the healthy (A)**

**and patient (B) groups – MNI coordinates**

|  | | **(A) Healthy** | | | | | **(B) Patients** | | | | |
| --- | --- | --- | --- | --- | --- | --- | --- | --- | --- | --- | --- |
| **Brain region** | **BA** | **MNI x** | **MNI y** | **MNI z** | **t(19)** | **Cluster size** | **MNI x** | **MNI y** | **MNI z** | **t(24)** | **Cluster size** |
| **HiGC approach > LoGC approach** | | | | | | | | | | | |
| **Frontal Lobe** | | | | | | | | | | | |
| L. Middle Frontal Gyrus (MFG) | 9 | -35 | 44 | 28 | 5.47 | 1170 | — | — | — | — | — |
| R. Middle Frontal Gyrus (MFG) | 9 | 36 | 41 | 30 | 5.79 | 1580 | — | — | — | — | — |
| L. Superior Precentral Sulcus (supPreCS) | 6 | -29 | -6 | 59 | 4.21 | 1990 | -30 | -3 | 60 | 4.52 | 1600 |
| R. Superior Precentral Sulcus (supPreCS) | 6 | 32 | -4 | 58 | 4.09 | 1745 | 32 | -5 | 60 | 4.91 | 1310 |
| **Occipital Lobe** | | | | | | | | | | | |
| L. Inferior Occipital Gyrus | 17 | -15 | -93 | -5 | 6.73 | 2580 | -16 | -95 | -7 | 6.82 | 2240 |
| R. Inferior Occipital Gyrus | 17 | 18 | -91 | -4 | 6.28 | 2470 | 20 | -92 | -4 | 7.14 | 1890 |
| **Temporal Lobe** | | | | | | | | | | | |
| L. Middle Temporal Gyrus (MTG) | 37 | -47 | -71 | 11 | 6.83 | 1875 | -46 | -72 | 11 | 6.01 | 1780 |
| R. Middle Temporal Gyrus (MTG) | 37 | 48 | -70 | 8 | 5.74 | 1660 | 48 | -71 | 11 | 5.93 | 1680 |
| **Parietal Lobe** | | | | | | | | | | | |
| L. Precuneus | 7 | -8 | -66 | 53 | 8.15 | 2560 | -10 | -61 | 54 | 8.24 | 2050 |
| R. Precuneus | 7 | 12 | -66 | 54 | 8.47 | 2340 | 9 | -60 | 57 | 8.03 | 2420 |
| L. Cuneus | 23 | -14 | -75 | 11 | 7.15 | 1270 | -12 | -76 | 11 | 7.61 | 1330 |
| R. Cuneus | 23 | — | — | — | 8.27 | 1360 | 15 | -73 | 10 | 7.96 | 1240 |
| **Sub-cortical Regions** | | | | | | | | | | | |
| L. Ventral Striatum (VS) |  | -16 | 5 | -3 | 5.17 | 350 | — | — | — | — | — |
| R. Ventral Striatum (VS) |  | 27 | 8 | -5 | 4.96 | 410 | 24 | 5 | -4 | 3.27 | 110 |
| L. Ventral Tegmental Area (VTA) |  | -11 | -15 | -9 | 4.76 | 370 | -13 | -16 | -9 | 3.02 | 100 |
| R. Ventral Tegmental Area (VTA) |  | 13 | -16 | -8 | 5.42 | 280 | 14 | -17 | -8 | 3.13 | 100 |
| Periaqueductal Gray (PAG) |  | 0 | -31 | -3 | 4.79 | 430 | -1 | -30 | -5 | 3.89 | 180 |
|  | | | | | | | | | | | |
| **LoGC approach > HiGC approach** | | | | | | | | | | | |
| **Frontal Lobe** | | | | | | | | | | | |
| L. Inferior Frontal Gyrus (IFG) | 9/46 | -45 | 23 | 21 | -4.85 | 1520 | -40 | 43 | -4 | -3.29 | 650 |
| R. Inferior Frontal Gyrus (IFG) | 9/46 | 44 | 23 | 21 | -4.68 | 1980 | 44 | 45 | -4 | -3.01 | 620 |
| Medial Prefrontal Cortex (mPFC) | 10 | 3 | 56 | 5 | -4.37 | 2470 | 5 | 60 | 5 | -4.18 | 1920 |
| R. Superior Fronta Gyrus (SFG) | 8 | 8 | 37 | 43 | -4.13 | 1340 | 6 | 39 | 41 | -3.86 | 690 |
| **Temporal Lobe** | | | | | | | | | | | |
| L. Inferior Temporal Gyrus (ITG) | 21 | -60 | -12 | -19 | -3.95 | 2030 | -59 | -10 | -17 | -3.28 | 1150 |
| R. Inferior Temporal Gyrus (ITG) | 21 | 63 | -15 | -20 | -3.76 | 2180 | 62 | -10 | -18 | -3.17 | 1875 |
| **Insular Cortex** | | | | | | | | | | | |
| L. Insula | 13 | -44 | 3 | -6 | -4.89 | 750 | — | — | — | — | — |
| R. Insula | 13 | 43 | 9 | -5 | -4.07 | 980 | 45 | 8 | -10 | -3.15 | 790 |
| **Cingulate Cortex** | | | | | | | | | | | |
| Anterior Cingulate | 32 | 1 | 41 | 15 | -4.98 | 1280 | -1 | 43 | 19 | -4.73 | 1140 |
| Posterior Cingulate | 29 | -2 | -39 | 23 | -4.26 | 1490 | 4 | -41 | 19 | -4.98 | 1080 |

MNI coordinates were converted from the original Talairach coordinates using the Lancaster et al. (2007) nonlinear transform as implemented in NiMARE v0.2 (tal2mni). For Talairach equivalents see Supplementary Table 1. BA, Brodmann area; L, left; R, right.
